# The effects of an innovative integrated care intervention in Brazil on local health service use by dependent older people

**DOI:** 10.1186/s12913-022-07552-y

**Published:** 2022-02-11

**Authors:** Peter Lloyd-Sherlock, Karla Giacomin, Lucas Sempé

**Affiliations:** 1 University of East Anglia, Norwich, England; 2 Oswaldo Cruz Foundation, Rio de Janeiro, Brazil

**Keywords:** older people, rehabilitation, planned visits, health policy in Brazil

## Abstract

**Background:**

Since 2011, the Brazilian city of Belo Horizonte has been operating an innovative scheme to support care-dependent older people in disadvantaged communities: Programa Maior Cuidado (PMC – Older Person’s Care Program). This paper examines two potential associations between inclusion in PMC on types of outpatient health service utilization by dependent older people. The first is that being in PMC is associated with a higher frequency of outpatient visits for physical rehabilitation. The second is that being in PMC is associated with a higher frequency of planned versus unplanned outpatient visits.

**Methods:**

We apply a quasi-experimental design to a unique set of health administrative data recording visits to outpatient health services. We focus on comparisons of the universe of visits, transformed to ratios of planned/unplanned visits and rehabilitation/other reasons for visiting the outpatient service. First, we preprocess our sample through different matching techniques such as ‘coarsened exact matching’ (CEM), ‘nearest neighbor’ based on logit scores (NN), ‘optimal pair’ (OP) and ‘optimal full’ (OF) methods. Second, we estimate marginal effects of being in PMC on our outcomes of interest. We use Poisson regressions controlling for individual and community factors and use robust standard errors. Our results are presented as the comparative incidence ratio of PMC on rehabilitation and planned visits.

**Results:**

We find significant positive incidence rates for belonging to PMC for both outcomes of interest under all matching specifications. Poisson models using CEM shows a higher incidence rate for planned visits in comparison to unplanned visits, 1.3 (95% CI 1.1–1.4), by PMC patients compared to the non-PMC controls, and a higher proportion of visits for rehabilitation, 3.4 (95% CI 1.7–6.8). Similar positive results are found across other matching methods and models.

**Conclusions:**

Our analysis reveals significant positive associations between older people included in PMC and a matched set of controls for a greater ratio of making outpatient visits that were planned, rather than unplanned. We find similar associations for the proportion of visits made for rehabilitation, as opposed to other reasons. These findings indicate that PMC influences some elements of outpatient health service utilization by dependent older people.

**Supplementary Information:**

The online version contains supplementary material available at 10.1186/s12913-022-07552-y.

## Background

In many countries, there are growing concerns about the inefficiency and rising costs of existing forms of health service provision for older adults with complex health and social care needs, and these have spurred the development of new interventions. Responses have included efforts to integrate health and social care and to refocus provision towards domiciliary settings [1–3]. Obtaining robust evidence about the effects of these interventions has been challenging, due to gaps in systematic data reporting and monitoring, as well as difficulties in establishing clear causal pathways related to outcomes of interest [4]. Also, the effects of such interventions are often strongly contingent upon the wider contexts in which they operate [5]. In less-developed world regions, which account for 70% of the global population aged 70 or more, health care resources are especially scarce increasing the need for effective models of care [6]. Although there are some published studies of integrated care interventions in these regions, these do not specifically relate to older people [7–9].

This paper focusses on an intervention offering community-based health and social care to care-dependent older people in the Brazilian city of Belo Horizonte. The intervention is jointly managed by local health and social assistance agencies and provides a number of support services for older people living in deprived neighborhoods of the city. These include daily visits and care support from trained lay carers who work closely with health and social work professionals. The paper examines associations between participation in the scheme and two elements of government health center outpatient service utilization: unplanned visits and rehabilitation. Unplanned outpatient visits are associated with less efficient use of resources and greater risk of emergency hospital admission [10]. Likewise, outpatient rehabilitation has been shown to be a cost-effective intervention, which can significantly lower hospital readmission risk [11–14]. Consequently, reducing the share of visits made on an unplanned basis and increasing the share of visits made for the purpose of rehabilitation are desirable policy outcomes.

### Intervention of interest: Programa Maior Cuidado

Like many countries, Brazil has separate national systems for health services, the Unified Health System (UHS), and for social care, the Unified System for Social Assistance (USSA). Management of both systems is mainly delegated to municipal governments [15]. The UHS has a strong focus on community-based family health provided by local health centers [16]. Nevertheless, the large bulk of UHS spending is devoted to hospital-based services [17]. The USSA manages a range of welfare benefits and social services through its own networks of local offices. Historically, both systems have only had limited focus on older people’s needs, as older people accounted for a relatively small proportion of the population, especially in poorer neighborhoods. Over the next 20 years, the proportion of Brazil’s population aged 70 or more will more than double: from 6.1% in 2020 to 12.6% in 2040 [6]. As a result, there are growing policymaker concerns both about meeting the needs of care-dependent older people more effectively and mitigating pressures on inpatient hospital services.

Since 2011, the city of Belo Horizonte has been operating an experimental scheme to support care-dependent older people in disadvantaged communities: Programa Maior Cuidado (PMC – Older Person’s Care Program). The city government had been concerned about the limited capacity and sometimes very low quality of care provided by local long-term care facilities and by evidence of rapidly growing numbers of care-dependent older people living in poor neighborhoods. Consequently, it was keen to develop a new model of community-based health and social care for these older people [18].

PMC was jointly developed by the UHS and USSA in Belo Horizonte and involves close collaboration between local health posts and social assistance centers. PMC shares some broad principles with interventions such as home-based primary health care and hospital at home [1, 2]. However, the specific form of the intervention and the context in which it operates are both different. First, the capacity of the existing health system, including its clinically trained personnel, to proactively address the needs of older people is much more limited than in high-income countries. In 2018, per capita health spending in Brazil was less than a tenth that of the USA [19]. Consequently, deploying multi-disciplinary teams of clinical and non-clinical professionals was an unfeasible objective. Instead, the main element of PMC home support is through lay carers, who are recruited from similar communities, provided basic training and paid a basic wage.

Each participating family receives between 10 and 40 h of help a week from a PMC carer, depending on the level of need of the older person and the family’s wider situation. PMC carers are not expected to replace family care responsibility for dependent relatives. Instead, the focus is on providing family carers some respite from what is often an exhausting 24/7 activity. PMC carers are also expected to work with families to build their care skills and competence, and to agree a care plan. As well as providing daily support, PMC carers monitor the situation of the older person and report back to monthly case reviews conducted by staff at health and social assistance posts.

Previous research on PMC has looked at its development and its operational processes [20]. It also includes qualitative evidence from interviews with local health and social assistance professionals indicating that PMC can enhance communication and engagement between home carers and local health services [21]. This study aims to extend this evidence base, through quantitative analysis with reference to two specific aspects of health service utilization.

The first issue of interest is whether being in PMC is associated with a higher frequency of outpatient visits for rehabilitation. Along with their other responsibilities, PMC carers continue to support older people when they are in hospital, with a view to facilitating discharge back to families and to enhance person-centered linkages between in- and out-patient providers [20]. This includes supporting recovery and reducing risk of readmission by identifying rehabilitation needs and reporting them to the PMC case reviews. The second issue is whether being in PMC is associated with a higher frequency of planned versus unplanned outpatient visits. PMC carers receive some basic trained to recognize warning signs of potential acute health problems and are required to report them immediately to health centers.

## Methods

### Data sources

Data on outpatient health service use comes from the Brazilian Health System (SUS) collected by the government of the city of Belo Horizonte (BH). This includes visits made by people aged 60 and over to the city’s 76 government public health posts. Data covers total number of visits during from April to June 2018.

BH also collects administrative data on PMC users. These records include users’ addresses, as well as their age and sex, and an individual patient number. The project team were granted ethical approval and access to anonymised patient data for the period April to June 2018 and is limited to the period due to administrative availability. Both data bases were linked through individual patient numbers. Due to the nature of the matching technique, we discard observations with missing covariates data. Discarded observations did not show outlier behaviours.

As both datasets do not include personal data on socio-economic or health status, we used patient addresses to construct a proxy indicator of socio-economic status based on a third data set for 275 micro-districts with median populations of 3827 (mean = 8546; standard deviation = 9752) produced by the public Institute for Applied Economic Research (Ipea) [22]. A limitation of this approach is that it assumes there is no significant socio-economic heterogeneity within each micro-district. To assess that limitation, we performed a kernel density spatial analysis of population access to public health centers.

### Outcome and covariate variables

In this study, we compare proportions of type of visits within a fixed universe of visits. We compare the ratio of planned/unplanned visits and the ration of rehabilitation visits/other reasons to visit the outpatient service. We study two outcome variables. The first is the ratio of outpatient visits for rehabilitation to all outpatient visits. The second variable is the ratio of planned outpatient visits versus unplanned ones.

The covariates used during the matching process are (i) at the individual level: sex, age, month of visit to the health care, household latitude and longitude, and distance to the health center; (ii) at the micro-district level, the social vulnerability index (SVI), the household economic dependency ratio (HEDR), life expectancy in years and household income per capita in Brazilian Reais. The SVI is a normalized composite index ranging from 0 to 1, based on 16 variables that reflect urban infrastructure, human capital, work and income. The HEDR is a ratio between the number of people in poor households where more than 50% of the household income comes from older people and the total population.

### Statistical methods

This is a cross-sectional quasi-experimental analysis of the effects of PMC on types of health service use. Our analytical model estimates the association of being in PMC on two outcomes: the likelihoods of planned outpatient visits and of outpatient visits for rehabilitation, compared to other types of outpatient visits. We apply a counterfactual framework to estimate an average treatment effect on the treated (ATT) of being in PMC for these outcomes of interest [23].

Due to the targeted nature of PMC and its focus on poorer neighborhoods, characteristics of relevance to our analysis were likely to differ between older people in PMC and the city’s general population, even after controlling for age and sex. Consequently, simple comparisons between older people enrolled in PMC and other older people are not valid without including mediating effects. To address that, we combine a matching method with a regression model [24] to address potential weak identification bias.

The first step implies preprocessing our sample with a ‘coarsened exact matching’ (CEM) technique. CEM involves partitioning covariates into groups -called bins- when their values are similar [25]. CEM then performs an exact matching of observations (treatment and control) so only units with identical coarsened covariates values are matched and the remaining observations are discarded. As a sensitivity analysis, we use other three matching strategies: the ‘nearest neighbor’ based on logit scores (NN), ‘optimal pairs’ (OP) and ‘optimal full’ (OF) [26]. Details of the matching methods and specifications used can be found in the appendix.

Our second step is computing PMC’s marginal effects on our outcomes of interest. We calculate the average treatment on the treated (ATT) effect as an incidence ratio (IR) since our variables are dichotomous. For that purpose, we conduct Poisson regressions in order to estimate the ATT effects of PMC on our variables of interest, such as in:

$$\log \left(\hat{y}\right)={\beta}_0+{\beta}_1\mathrm{PMC}+\epsilon$$

Where *β*_1_ represents the coefficient associated with belonging to PMC on the outcome variable $\hat{y}$. Alpha levels are set at .05. Exponenciating the coefficient *β*_1_ gives us the incidence rate associated to belonging to PMC in comparison to not belonging to the program, with relation to the outcome variables. We use robust standard errors (for CEM) and cluster-robust standard errors (for NN, OP, OT) to address heteroskedasticity. We use regression weights for CEM and OF to balance the covariates in the regression models. In the cases of NN and OP, weights equal to the number of observations are drawn for unused PMC and non-PMC observations.

Additionally, as a robustness test, we conduct Log binomial regressions using the same specifications detailed above.

## Results

**Fig. 1:**
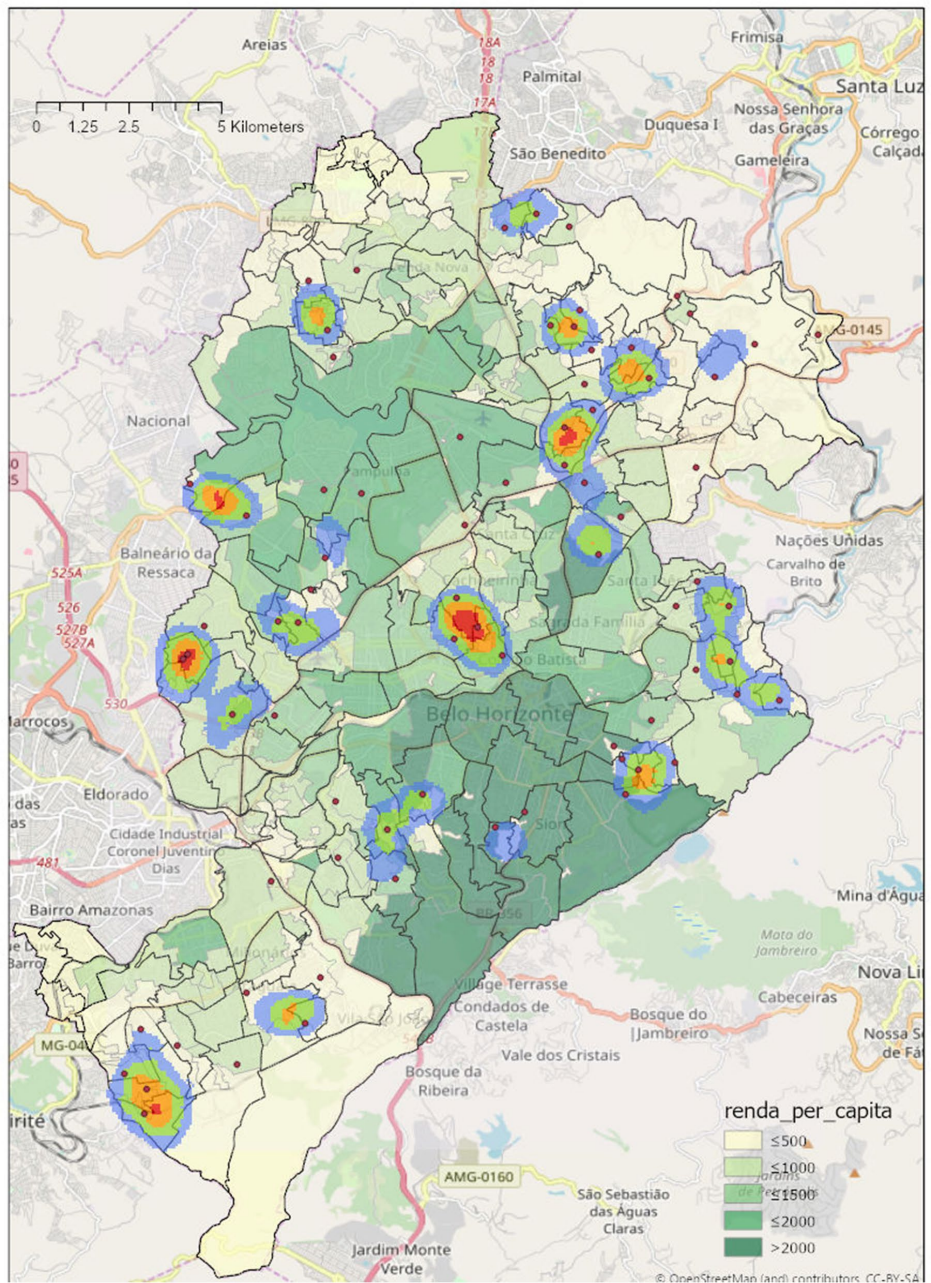
Density of residential segregation by people over 60 using government health posts (red dots) from April to June 2018 in the city of Belo Horizonte. *Note*: Legend: Micro-districts average income per capita (in Brazilian Reais) ≤500 (£90); ≤1000 (£180); ≤1500 (£270); ≤2000 (£360); > 2000 (£360)

Figure 1 shows the results of our preliminary a kernel density spatial estimation of health post utilization of people aged 60 or more. The location of these health posts and utilization of health services were both heavily concentrated in low-income neighborhoods, represented by the lighter green polygons in the map (less than 500 Brazilian Reais per capita monthly income, which is equivalent to less than £90 in 2018 using an annual average exchange rate).

As part of planning our research design we discussed these patterns with local experts who informed us that the low number of visits from more affluent Belo Horizonte neighborhoods is likely to reflect a higher rate of private health service utilization by older people in those areas, in line with evidence from other studies that rich older Brazilians mainly use private health care providers [27]. This suggests a high level of socioeconomic segregation, where socio-economic differentials between PMC and non-PMC users are likely to be smaller than they would be if health services utilization were more evenly spread across different neighborhoods. Additionally, key informants from the city validated this assumption of socioeconomic spatial homogeneity and a similar level of spatial socio-economic sorting has been observed for other cities in Brazil [28].

From the start of April 2018 to the end of June 2018 the average total number of visits by people aged 60 or more to each of the 76 health posts was 1150.7 (standard deviation (sd) = 920.61). Across all 76 posts, there were 87,455 visits by people aged 60 or more, involving 24,554 different individuals. As such, each of these older individuals made on average 3.52 visits over the three-month period sd = 3.25). Conversely, the data indicate that the large majority (92%) of people aged 60 or more living in the municipality (302,174, based on Census 2010)^1^ made no visits to a government health post over this period. Of those older people who made at least one visit to a health post over this period, 366 were enrolled in PMC, representing 19% of its membership.

Before the matching process, PMC users had an average of 1.29 (sd = .53) rehabilitation sessions while non-PMC had 1.48 (sd = .78), and PMC users had an average of 2.58 (sd = 1.8) planned visits while non-PMC had 2.11 (sd = 1.67). We observe no differences in the types of outpatient services offered by the same health posts to the PMC and the control groups.

Tables 1 and 2 show summary statistics for unmatched data and CEM for planned visits and rehabilitation, respectively. We observe, in both unmatched datasets, that PMC users are on average 10 years older than non-PMC users and the proportion of women is 5 to 6% higher. PMC users tend to live in more deprived areas in terms of lower per capita income, with lower life expectancy and more household economic dependency on older members.^2^

**Table 1:** Descriptive statistics - Planned: Unmatched and CEM

Characteristic	Unmatched			CEM		
	Non PMC, *N* = 59,812^1^	PMC, *N* = 1317^1^	*p*-value^2^	Non PMC, *N* = 4727^1^	PMC, *N* = 909^1^	Standardised mean difference
Individual variables						
Female	19,487 (33%)	373 (28%)	0.001	874 (18%)	208 (23%)	<.0001
Age	68 (64, 75)	79 (71, 85)	< 0.001	70 (65, 77)	76 (70, 82)	0.013
Month of visit			0.4			
April	21,498 (36%)	489 (37%)		1842 (39%)	347 (38%)	<.0001
June	17,134 (29%)	355 (27%)		1183 (25%)	238 (26%)	<.0001
May	21,180 (35%)	473 (36%)		1702 (36%)	324 (36%)	<.0001
Latitude	609,455 (604,703, 612,913)	609,813 (605,439, 612,932)	0.4	610,177 (603,614, 612,806)	610,174 (605,507, 613,770)	<.0001
Longitude	7,799,066 (7,795,080, 7,803,396)	7,798,510 (7,794,117, 7,803,848)	0.001	7,798,081 (7,788,783, 7,805,845)	7,798,454 (7,794,381, 7,804,153)	−0.004
Distance from household to health centre	420 (280, 578)	408 (267, 567)	0.056	409 (279, 575)	394 (257, 546)	<.0001
% Planned visit (Yes)	26,786 (45%)	695 (53%)	< 0.001	2270 (48%)	495 (54%)	
Micro-district variables						
Social vulnerability Index (SVI)	0.44 (0.38, 0.49)	0.45 (0.42, 0.49)	< 0.001	0.49 (0.43, 0.49)	0.45 (0.42, 0.52)	<.0001
Household economic dependency on elderly (HEDR)	1.54 (0.89, 1.85)	1.62 (1.17, 1.86)	< 0.001	1.62 (0.86, 1.92)	1.62 (1.16, 1.86)	<.0001
Life expectancy (in years)	71.38 (69.20, 73.42)	70.79 (68.50, 72.76)	< 0.001	69.20 (68.47, 71.42)	70.53 (68.47, 72.40)	<.0001
Income per capita (in Brazilian reais)	419 (318, 556)	373 (305, 504)	< 0.001	318 (299, 423)	359 (299, 463)	<.0001

^1^n (%); Median (IQR)

^2^Pearson’s Chi-squared test; Wilcoxon rank sum test

**Table 2:** Descriptive statistics - Rehabilitation: Unmatched and CEM

Characteristic	Unmatched			CEM		
	Non PMC, *N* = 26,138^1^	PMC, *N* = 510^1^	*p*-value^2^	Non PMC, *N* = 1333^1^	PMC, *N* = 325^1^	Standardised mean difference
Individual variables						
Female	8355 (32%)	133 (26%)	0.005	249 (19%)	70 (22%)	<.0001
Age	68 (64, 75)	78 (70, 84)	< 0.001	71 (66, 76)	73 (68, 80)	−0.002
Month of visit			> 0.9			
April	9462 (36%)	186 (36%)		553 (41%)	117 (36%)	<.0001
June	7384 (28%)	141 (28%)		272 (20%)	87 (27%)	<.0001
May	9292 (36%)	183 (36%)		508 (38%)	121 (37%)	<.0001
Latitude	609,445 (604,527, 612,848)	609,803 (605,519, 612,902)	0.4	609,499 (602,543, 612,915)	610,428 (605,507, 613,844)	−0.003
Longitude	7,798,942 (7,795,095, 7,802,850)	7,799,000 (7,794,381, 7,805,355)	0.7	7,798,390 (7,788,804, 7,801,427)	7,799,079 (7,794,637, 7,805,377)	0.004
Distance from household to health centre	419 (279, 578)	408 (275, 567)	0.3	421 (293, 599)	435 (287, 548)	<.0001
% Rehabilitation visit (Yes)	616 (2.4%)	71 (14%)	< 0.001	23 (1.7%)	34 (10%)	
Micro-district variables						
Social vulnerability Index (SVI)	0.43 (0.39, 0.49)	0.44 (0.40, 0.49)	< 0.001	0.44 (0.40, 0.49)	0.44 (0.40, 0.49)	<.0001
Household economic dependency on elderly (HEDR)	1.46 (0.88, 1.85)	1.62 (1.16, 1.93)	< 0.001	1.46 (0.83, 1.84)	1.54 (0.86, 1.86)	<.0001
Life expectancy (in years)	71.38 (69.20, 73.42)	70.79 (68.50, 72.76)	< 0.001	71.08 (69.20, 73.28)	71.01 (68.50, 72.80)	<.0001
Income per capita (in Brazilian reais)	419 (318, 556)	373 (292, 504)	< 0.001	406 (318, 549)	402 (299, 510)	<.0001

^1^n (%); Median (IQR)

^2^Pearson’s Chi-squared test; Wilcoxon rank sum test

The CEM technique retrieved 325 and 1333 observations in the treatment and control groups for rehabilitation and 909 and 4727 observations for planned visits. After matching, all standardized mean differences for the covariates were below .03. Also, all absolute within-pair differences of each covariate were below .13 for both outcomes, suggesting an adequate balance between groups.

The NN, generated 465 and 1243 observations in each group for rehabilitation and planned visits, respectively. The OP generated 510 and 1317 observations. The OF generated 465 and 1317 treatment observations, along with 26,138 and 59,812 control observations. Descriptive statistics are found in the appendix. Sensitivity tests (*Γ*) for NN and OP became significant in a range of 1.4 and 1.7 for rehabilitation visits and 1.1 and 1.4 for planned visits. This suggests a higher sensitivity to hidden bias in the case of the matched rehabilitation visits. Tables S1 and S2 in the Appendix present results for the other matching strategies.

### Main results

Table 3 presents the adjusted marginal associations between being in PMC and making a health post visit for making a planned visit in comparison to an unplanned one is significant in all three models for all matching specifications. In all cases we find a significant positive incidence rate, whereby the CEM shows a higher incidence rate ratio in comparison to the control group (1.3, 95% CI 1.1–1.4). The CEM shows belonging to PMC increases the likelihood that outpatient visits were made on a planned rather than an unplanned basis. Results for other matching techniques are the following: NN: 1.1 (95% CI 1–1.3), OP: 1.1 (95% CI 1–1.3), OT: 1.2 (95% CI 1.1–1.3).

**Table 3:** Poisson regression parameters – Ratio of planned visits

Parameter	CEM		NN		OP		OT	
	Incidence Rate	95% CI	Incidence Rate	95% CI	Incidence Rate	95% CI	Incidence Rate	95% CI
PMC	1.3 ***	(1.1–1.4)	1.1 **	(1–1.3)	1.1 **	(1–1.3)	1.2 ***	(1.1–1.3)
Sex (Female)	1.1 **	(1–1.3)	1	(0.9–1.1)	1	(0.9–1.2)	1	(1–1)
Age	1	(1–1)	1	(1–1)	1	(1–1)	1 **	(1–1)
SVI	0 **	(0–0.8)	0	(0–1.5)	0	(0–1.2)	0 ***	(0–0)
HEDR	1.3 **	(1–1.6)	1.4 **	(1.1–1.7)	1.4 **	(1.1–1.8)	1.4 ***	(1.4–1.5)
Income per capita	1	(1–1)	1	(1–1)	1	(1–1)	1 ***	(1–1)
Life expectancy	0.9	(0.9–1)	0.9	(0.9–1)	0.9	(0.9–1)	0.9 ***	(0.9–1)
Latitude	1 **	(1–1)	1	(1–1)	1	(1–1)	1	(1–1)
Longitude	1	(1–1)	1	(1–1)	1	(1–1)	1	(1–1)
Month: June (ref. = April)	1	(0.9–1.1)	1	(0.9–1.2)	1	(0.9–1.2)	1	(0.9–1)
Month: May (ref. = April)	1	(0.9–1.1)	1	(0.9–1.1)	1	(0.9–1.1)	1	(0.9–1)
*Fixed effects (Health posts)*	*Yes*		*Yes*		*Yes*		*Yes*	

Note: *** p < .001, ** p < .05, * p < .01

Table 4 presents the adjusted marginal associations between being in PMC and making a rehabilitation visit compared to other reasons under all matching techniques. This demonstrates that being in the PMC group was associated with a higher likelihood (3.4, 95% CI 1.7–6.8) that outpatient visits were made for rehabilitation rather than for other reasons. Results for other matching techniques are the following: NN: 2.1 (95% CI 1.3–3.5), OP: 2 (95% CI 1.3–3), OT: 2.5 (95% CI 2–3.2).

**Table 4:** Poisson regression parameters – Ratio of rehabilitation visits

Parameter	CEM		NN		OP		OT	
	Incidence Rate	95% CI	Incidence Rate	95% CI	Incidence Rate	95% CI	Incidence Rate	95% CI
PMC	3.4 ***	(1.7–6.8)	2.1 **	(1.3–3.5)	2 **	(1.3–3)	2.5 ***	(2–3.2)
Sex (Female)	1.7	(0.7–4.5)	1.4	(0.8–2.5)	1.1	(0.7–1.7)	1.3 **	(1.1–1.5)
Age	1.1 **	(1–1.1)	1	(1–1)	1 **	(1–1)	1 ***	(1–1)
SVI	0	(0–143,533)	0 **	(0–0)	0	(0–20.2)	0 ***	(0–0)
HEDR	346.5 **	(4.7–25,676)	3.7 **	(1.5–9.4)	2.6 **	(1.1–6.4)	6.4 ***	(4.8–8.5)
Income per capita	1	(1–1)	1	(1–1)	1	(1–1)	1	(1–1)
Life expectancy	0.7	(0.3–1.9)	1	(0.7–1.3)	0.9	(0.7–1.2)	0.7 ***	(0.7–0.8)
Latitude	1	(1–1)	1	(1–1)	1	(1–1)	1	(1–1)
Longitude	1 **	(1–1)	1	(1–1)	1	(1–1)	1 ***	(1–1)
Month: June (ref. = April)	1.3	(0.5–3.4)	1.1	(0.6–1.9)	0.9	(0.5–1.4)	1	(0.9–1.2)
Month: May (ref. = April)	0.4 **	(0.2–0.9)	0.8	(0.5–1.4)	0.8	(0.5–1.3)	1	(0.8–1.1)
*Fixed effects (Health posts)*	*Yes*		*Yes*		*Yes*		*Yes*	

Note: *** p < .001, ** p < .05, * p < .01

Similar results are found for models using Log Quasibinomial. Coefficients are reported in Tables S1 and S2 in the Appendix.

## Discussion

### Statement of main findings

Our two-step analysis shows a significant association for older people being included in the PMC program and making visits to government health posts on a planned rather than unplanned basis, when compared to a matched set of people not in PMC. Applying the same analytical method, we find being included in PMC was significantly associated with a higher proportion of rehabilitation visits to health posts, as opposed to other motives for visits.

### Limitation of the study

Our study design has several limitations. First, the scarcity of individual-level data does not permit us to identify potential predictors of service use. To compensate for this, we use a rich set of neighborhood-level data. Second, although matching techniques enable balancing of observations based on observed covariates, unmeasured confounding variables may still be present in our analysis. We address any hidden bias through sensitivity tests and comparing post-match covariates between groups, and these indicate that the estimates of treatment effects we report are robust. Third, in neither the PMC nor the control groups do we observe any patients making many visits, which might have biased our estimates. As a robustness test, we performed the same analysis excluding people making more than four and five visits. The number of such people was very small and so the findings were almost identical to those of our analysis.

Using a complete dataset by discarding observations with missing data on covariates is done to prevent introducing bias on the matching process. As matching implies finding similar observations based on covariates, the lack of data relies on an assumption of non-relevance of that piece of information. As results are robust modelling with complete dataset, and discarded observations were not outliers, our approach is avoids introducing bias to the analysis.

Finally, we dismiss possible spillover effects over the health and care systems, which could occur under the scarcity of the limited resources available on outpatient health services. This is based on previous research that does not suggest substitution of services in LMICs [30], which was confirmed by our local stakeholders.

### Comparisons to other studies

Our descriptive finding that 92% of people aged 60 or more in Belo Horizonte made no visit to a government health post between April and June 2018 indicates older people’s engagement with the city’s supposedly universal public primary health care system was limited. This finding does not match the results of other studies in Brazil, which report higher rates of health post utilization by people at older ages [31]. For example, a national survey of Brazilians aged 50 and over reported that 63.5% had made at least one visit to a government health post during the previous year [32]. One explanation for the lower rate of health post utilization reported in our study is that it refers to a three-month period rather than 12 months. Some specific local factors may also be relevant. For example, Belo Horizonte has a much hillier terrain than most Brazilian cities and its poorer neighborhoods are characterized by very steeply sloping streets, creating specific difficulties for accessing services for older people with limited mobility and who lack private transport.

Studies about the frequency and determinants of outpatient health service use by older people in Brazil and other countries do not look at the same outcomes of interest as in our study. With reference to the UK [33], it has been observed that: “there has been little attention to acuity of presentation to GPs during the working week, and in particular, multi-morbid community-dwelling older person’s utilization of planned and unplanned GP care.” Consequently, it is not possible to make direct comparisons between our two main findings and the wider literature.

Our findings accord with qualitative research on PMC which indicates participation in the programme promotes effective engagement with local health services [20]. The unusual nature of PMC limits comparisons with interventions involving teams of clinical and non-clinical professionals, such as home-based primary health care or hospital at home [34]. PMC’s use of lay carers shares elements with initiatives promoting community-based support for dependent older people in other developing countries [35]. However, these schemes rely on volunteers rather than paid carers and have not been subject to quantitative analysis.

### Meaning of this study for policy

This study provides some evidence that interventions like PMC are associated with a more efficient use of scarce health resources. Participation in PMC is associated with a higher share of outpatient visits for the purpose of rehabilitation. This is in keeping with Brazil’s national health system protocol that primary health care providers should have lead responsibility for identifying and managing adult rehabilitation needs [29]. Leading causes of hospitalization of older people in Brazil include hip fracture and stroke, and studies in other countries demonstrate the benefits of outpatient rehabilitation for these conditions [36, 37]. They also demonstrate the potential cost savings from outpatient rehabilitation. For example, analysis of average monthly post-stroke care costs in the USA found that services provided in outpatient settings cost less than a sixth of those provided as inpatient services [1, 13] . When provided in the home setting as part of an inter-disciplinary intervention, this can reduce the need for inpatient hospital care.

This study finds an association between participation in PMC and a lower share of outpatient visits that were unplanned. This is likely to enhance both the technical and allocative efficiency of health services. A UK official review found that growing utilization of urgent and emergency outpatient care is leading to mounting costs and increased pressure on resources [38].

### Unanswered questions and further research

Key related areas for future research fall into two broad areas. First, there is an urgent need to identify and categorize other examples of interventions and policy experimentation that share some elements with PMC. This will establish the degree to which PMC is a unique experience or is representative of wider policy trends in Brazil and beyond.

Second, there is a need to develop comparative evidence about the effects of these different interventions. Doing so will be vital for addressing pressures on health services resulting from population ageing, the COVID-19 pandemic and fiscal austerity. Currently, this comparative evidence remains very limited [4, 34, 39, 40]. Our findings contribute to that evidence base, with reference to a specific set of effects for a single intervention. Research from Brazil, the UK and other countries shows that inadequate social care for older people in the community can contribute significantly to otherwise avoidable hospital and care home admissions [41–44]. We were unable to explore whether PMC does this.

## Conclusions

This study provides evidence that integrated care interventions for dependent older people can influence patterns of outpatient health service utilization in ways that can be considered both efficiency-enhancing and, also, beneficial to these older people. The range of health service utilization effects analyzed was limited by the available data, and the results we report may be specific to the detailed design of this intervention and the context in which it has been implemented. This demonstrates an urgent need for wider evaluations of these effects, including on utilization of inpatient care. Notwithstanding their limitations, our findings show the potential value of community-based interventions like PMC, as part of new models of integrated health and social care for dependent older people in poor settings.

## Supplementary Information

**Additional file 1: Table S1**. Log quasibinomial regression parameters – Ratio of planned visits. **Table S2**. Log quasibinomial regression parameters – Ratio of rehabilitation visits.

## Data Availability

The datasets generated and/or analysed during the current study are not publicly available due to confidentiality, but are available from the corresponding author on reasonable request.

## Footnotes

1 https://censo2010.ibge.gov.br/ (retrieved: 26/03/2021)

2 [89.2% of those aged over 65 received a social security or social assistance pension, while in a quarter (24,9%) of total households, older people contributed more than 50% of the household income [29].]
