# Supplementary material for: The effects of an innovative integrated care intervention in Brazil on local health service use by dependent older people
Source: BMC Health Serv Res. 2022 Feb 11;22:176. doi: 10.1186/s12913-022-07552-y (PMC8831035; doi:10.1186/s12913-022-07552-y)
Supplement: Supplementary file 1 — Additional file 1: Table S1. Log quasibinomial regression parameters – Ratio of planned visits. Table S2. Log quasibinomial regression parameters – Ratio of rehabilitation visits. [file 12913_2022_7552_MOESM1_ESM.docx]

Appendix

Table S1: Log quasibinomial regression parameters – Ratio of planned visits

|  | **CEM** | | **NN** | | **OP** | | **OT** | |
| --- | --- | --- | --- | --- | --- | --- | --- | --- |
| **Parameter** | **Incidence Rate** | **95% CI** | **Incidence Rate** | **95% CI** | **Incidence Rate** | **95% CI** | **Incidence Rate** | **95% CI** |
| PMC | 1.1 *** | (1.1 - 1.2) | 1.1 *** | (1.1 - 1.2) | 1.1 ** | (1 - 1.2) | 1.1 *** | (1 - 1.1) |
| Sex (Female) | 1.1 ** | (1 - 1.2) | 1 | (0.9 - 1.1) | 1 | (0.9 - 1.1) | 1 | (1 - 1) |
| Age | 1 | (1 - 1) | 1 | (1 - 1) | 1 | (1 - 1) | 1 *** | (1 - 1) |
| SVI | 0.1 ** | (0 - 0.6) | 0.1 ** | (0 - 0.8) | 0.1 | (0 - 1.2) | 0.1 *** | (0 - 0.1) |
| HEDR | 1.1 | (1 - 1.3) | 1.3 *** | (1.1 - 1.5) | 1.3 *** | (1.1 - 1.5) | 1.2 *** | (1.2 - 1.3) |
| Income per capita | 1 | (1 - 1) | 1 | (1 - 1) | 1 | (1 - 1) | 1 *** | (1 - 1) |
| Life expectancy | 0.9 ** | (0.9 - 1) | 1 | (0.9 - 1) | 1 | (0.9 - 1) | 1 *** | (1 - 1) |
| Latitude | 1 ** | (1 - 1) | 1 | (1 - 1) | 1 | (1 - 1) | 1 | (1 - 1) |
| Longitude | 1 | (1 - 1) | 1 | (1 - 1) | 1 | (1 - 1) | 1 | (1 - 1) |
| Month: June (ref. = April) | 1 | (0.9 - 1.1) | 1 | (0.9 - 1.1) | 1 | (0.9 - 1.1) | 1 ** | (1 - 1) |
| Month: May (ref. = April) | 1 | (1 - 1.1) | 1 | (0.9 - 1.1) | 1 | (0.9 - 1.1) | 1 | (1 - 1) |
| *Fixed effects (Health posts)* | *Yes* | | *Yes* | | *Yes* | | *Yes* | |
|  |  |  |  |  | *Note: *** p <.001, ** p <.05, * p <.01* | | | |

Table S2: Log quasibinomial regression parameters – Ratio of rehabilitation visits

|  | **CEM** | | **NN** | | **OP** | | **OT** | |
| --- | --- | --- | --- | --- | --- | --- | --- | --- |
| **Parameter** | **Incidence Rate** | **95% CI** | **Incidence Rate** | **95% CI** | **Incidence Rate** | **95% CI** | **Incidence Rate** | **95% CI** |
| PMC | 2.5 *** | (1.9 - 3.2) | 2.1 *** | (1.6 - 2.8) | 1.8 *** | (1.4 - 2.3) | 1.5 *** | (1.3 - 1.8) |
| Sex (Female) | 1.7 ** | (1.1 - 2.7) | 1.5 *** | (1.2 - 2) | 1.1 | (0.9 - 1.3) | 1.2 *** | (1.1 - 1.3) |
| Age | 1 *** | (1 - 1.1) | 1 | (1 - 1) | 1 *** | (1 - 1) | 1 *** | (1 - 1) |
| SVI | 0 ** | (0 - 0) | 0 *** | (0 - 0) | 0 ** | (0 - 0.2) | 0 *** | (0 - 0) |
| HEDR | 82.2 *** | (13.2 - 512.9) | 3.8 *** | (2.5 - 5.8) | 2.2 *** | (1.6 - 3.2) | 2.8 *** | (2.4 - 3.3) |
| Income per capita | 1 | (1 - 1) | 1 | (1 - 1) | 1 | (1 - 1) | 1 *** | (1 - 1) |
| Life expectancy | 0.7 | (0.5 - 1.2) | 1 | (0.8 - 1.2) | 0.9 | (0.8 - 1.1) | 0.9 ** | (0.9 - 1) |
| Latitude | 1 | (1 - 1) | 1 *** | (1 - 1) | 1 *** | (1 - 1) | 1 ** | (1 - 1) |
| Longitude | 1 *** | (1 - 1) | 1 | (1 - 1) | 1 ** | (1 - 1) | 1 *** | (1 - 1) |
| Month: June (ref. = April) | 1.4 | (1 - 1.9) | 1.2 | (0.9 - 1.5) | 0.8 | (0.6 - 1.1) | 1.1 | (1 - 1.2) |
| Month: May (ref. = April) | 0.5 *** | (0.3 - 0.7) | 1 | (0.9 - 1.1) | 0.9 | (0.8 - 1.1) | 1 | (0.9 - 1.1) |
| *Fixed effects (Health posts)* | *Yes* | | *Yes* | | *Yes* | | *Yes* | |
|  |  |  |  |  | *Note: *** p <.001, ** p <.05, * p <.01* | | | |
